# International survey and surgeon’s preferences in diagnostic work-up towards treatment of anterior shoulder instability

**DOI:** 10.1007/s00402-016-2443-7

**Published:** 2016-03-14

**Authors:** Hanneke Weel, Wouter Tromp, Peter R. Krekel, Pietro Randelli, Michel P. J. van den Bekerom, Derek F. P. van Deurzen

**Affiliations:** Department of Orthopaedics and Traumasurgery, Onze Lieve Vrouwe Gasthuis, Oosterpark, 91091 AC Amsterdam, The Netherlands; Clinical Graphics, Delft, The Netherlands; Department of Orthopaedics, Academic Medical Center, Meibergdreef 9, 1105 AZ Amsterdam, The Netherlands; BovenIJ Hospital, Amsterdam, The Netherlands; 2nd Department of Orthopaedics, IRCCS Policlinico San Donato, Università degli Studi di Milano, Via Mangiagalli 30, San Donato Milanese, 20097 Milan, Italy

**Keywords:** Recurrence, Anterior instability, Shoulder joint, Diagnostic, Survey

## Abstract

**Purpose:**

Recurrent anterior shoulder instability after surgical treatment can be caused by bony defects. Several diagnostic tools have been designed to measure the extent of these bony lesions. Currently, there is no consensus which measurement tool to use and decide which type of surgery is most appropriate. We therefore performed an evaluation of agreement in surgeons’ preference of diagnostic work-up and surgical treatment of anterior shoulder instability.

**Methods:**

An international survey was conducted amongst orthopaedic shoulder surgeons. The survey contained questions about surgeons’ experience, clinical and radiological examination and the subsequent treatment for anterior shoulder instability. Descriptive statistics were used to present the data, and percentages of responding surgeons were calculated.

**Results:**

The questionnaire was completed by 197 delegates from 46 countries. 55 % of the respondents think evidence in current literature is sufficient on diagnostic work-up for anterior shoulder instability. Anamnestic, number of dislocations was most frequently asked (by 95 % of respondents), the most frequently used test is the apprehension test (91 %). For imaging, conventional X-ray in various directions was most performed, followed by MR arthrography and plane CT scan respectively. The responding surgeons perform surgery (labrum repair or Latarjet) in 51 % of the patients. A median of 25 % glenoid bone loss was given by the respondents, as cut-off from when to perform a bony repair.

**Conclusion:**

Many different diagnostic examinations for assessing shoulder instability are used and a high variety is seen in the use of diagnostic tools. Also no consensus is seen in the use of different surgical options (arthroscopic and open procedures). This implies the need for more research on diagnostic imaging and the correlation with specific subsequent surgical treatment.

**Level of evidence:**

Survey, level of evidence IV.

## Introduction

Post-traumatic anterior glenohumeral instability is the most common type of shoulder instability with a reported prevalence of 2 % [10]. During dislocation of the shoulder damage may occur to capsule, ligaments, labrum or bony structures such as the glenoid rim and humeral head [8]. The extent to which these bony defects of the glenoid rim occur is variable [5, 22] and so is the location and size of the Hill Sachs defect [3, 5]. Currently it is believed that recurrent instability is contributed by interplay of these existing bony defects [5, 25, 28].

Several methods have been developed to quantify the position and size of bony defects [22, 28] to predict the risk of recurrence [2]. Depending on this preoperative (or intra-operative) quantification the type of surgical treatment is chosen. Different surgical approaches have been proposed, each specifically aiming at correction of one or more of these defects. Although arthroscopic Bankart repair has evolved to a technically feasible procedure with minimal co-morbidity, relatively high recurrence rates have been published [9, 12, 26]. The traditional bony procedures such as these according to Latarjet have been reported to have lower recurrence rates [9, 12]. However, these procedures might have higher complication risks and also are not without failures [6]. In the last decade there have been an increasing number of reports on bony procedures performed in an arthroscopic fashion [4, 7]. However, current literature is still inconclusive when, with what amount of bone loss, to perform a soft tissue repair or a bony procedure.

To determine in which way orthopaedic surgeons assess shoulder instability and on what basis they choose for a specific therapeutic strategy, an international survey on clinical management of anterior shoulder instability was held at the European Society of Sports Traumatology, Knee Surgery and Arthroscopy (ESSKA) congress 2014. The aim of the survey is to investigate opinions of specialists in shoulder surgery on clinical decision-making when facing patients with anterior shoulder instability. The hypothesis is that there is a wide variation in clinical practice concerning diagnosing and treatment of anterior shoulder instability.

## Materials and methods

An expert team of two senior orthopaedic shoulder surgeons, one PhD candidate and one technical engineer, conducted an English questionnaire about anterior shoulder instability. The questionnaire was about the use of preoperative clinical examination, radiological assessment and treatment strategy of anterior shoulder instability. The questionnaire consisted of thirteen questions of which three open and ten multiple choice questions divided in three subtopics: clinical assessment, imaging and therapeutic management in anterior shoulder instability. Distributed questionnaire is added in Appendix.

The survey was distributed during the ESSKA congress in Amsterdam in May 2014. Paper questionnaires were distributed during and after sessions concerning shoulder instability related topics.

Notable strings were reported. Categorical data and dichotomous variables were summarized as percentages of the responding surgeons.

## Results

Of the surgeons participating in the shoulder lecture during the 9th ESSKA congress, 197 delegates from 46 different countries completed the survey (Table 1). Most (59 %) of the surgeons were from Europe. The median number of shoulder instability patients seen per surgeon per year was 50 ranging from 5 to 350 patients. Hereof, a median of 20 patients (range 0–200) was surgically treated.

**Table 1:** Respondent characteristics

		*N* (%)
Country	Europe	117 (59) West (55), East (45)
	Russia	4 (2)
	Middle east	32 (16)
	Asia	17 (9)
	Other (i.e. South Africa, South America)	23 (12)
	Unknown	4 (2)
ASI patients per year	<20	27 (14)
	20–50	71 (36)
	50–100	58 (29)
	>100	41 (21)

*ASI* anterior shoulder instability, *N* number, *%* percentage

### Assessment of the presence of anterior shoulder instability

**Fig. 1:**
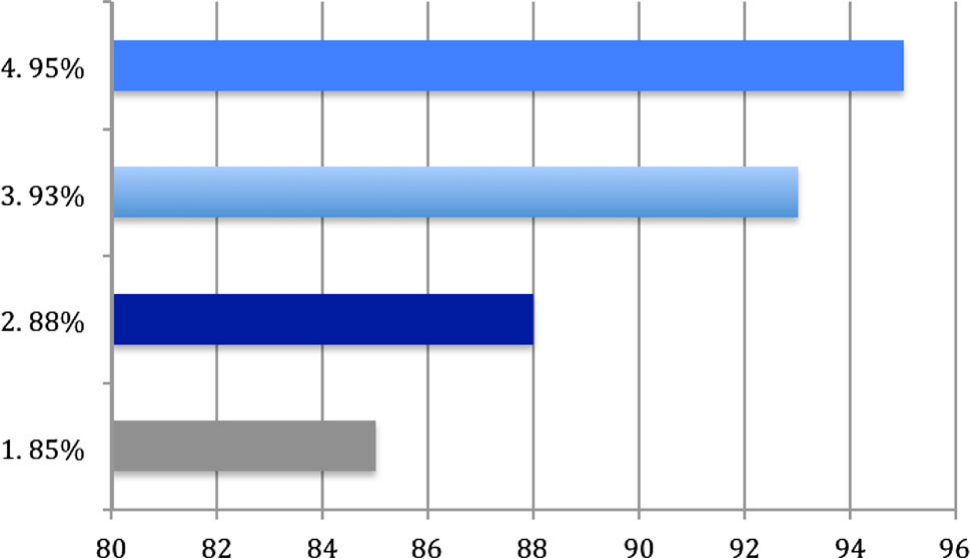
Most asked patient characteristics. 1 Profession, 2 performing sports, 3 traumatic event, 4 number of dislocations

During patient evaluation in the outpatient clinic, most frequently evaluated patient characteristics by the surgeons are shown in graphically shown in Fig. 1.

**Fig. 2:**
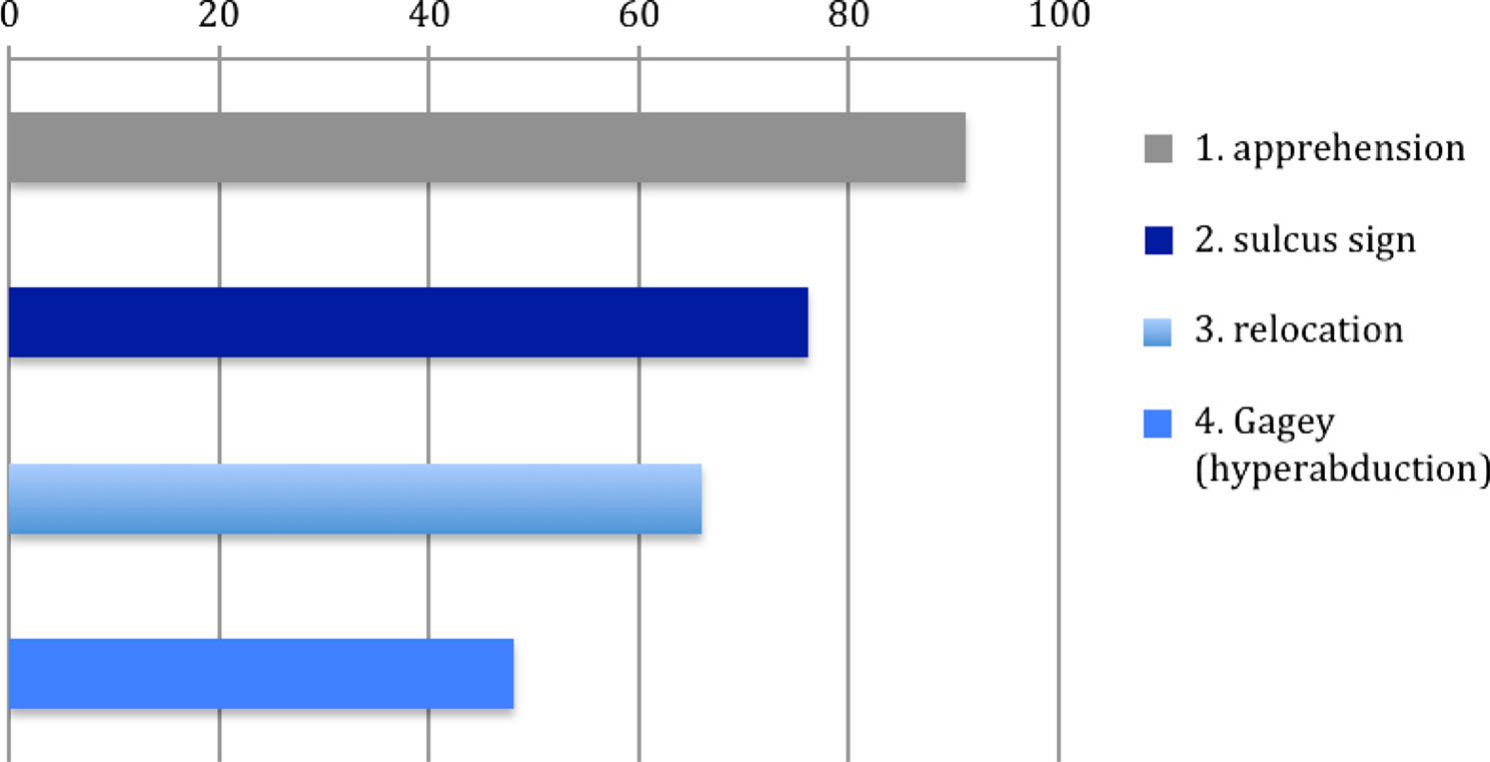
Most performed physical examination tests given in percentages (%)

During physical examination, the majority of surgeons perform the sulcus sign test (76 %), apprehension test (91 %) and relocation test (66 %). The hyperabduction test of Gagey is less frequently used (48 %), see Fig. 2.

### Shoulder instability and imaging

Conventional X-rays were taken anterior posterior (65 % of all respondents) and/or the Bernageau view (56 %) and less often the Velpeau view (11 %). The MR arthrography was used by 54 % respondents, plane CT scan was used by 27 % respondents. Imaging modalities were used by 65 % of surgeons to quantify the Hill Sachs defect, 85 % of the respondents measure glenoid bone loss with either plane MR (26 %) and/or CT scan (61 %). Nine respondents (5 %) use both MR and CT scan for measuring bone loss. A total of 17 % of respondents do not measure bone loss. Different methods of calculation methods are used to assess percentage of bone loss: Sugaya [22] is used in 39 %, the Index X [17] in 22 %, ITOY [11] in 21 % and Hardy [19] in 14 %.

### Therapeutic management of shoulder instability

About half of the respondents (55 %) think sufficient knowledge and evidence on the decision method for an operative treatment of shoulder instability is available in current literature and guidelines. Approximately half of the patients seen in clinic with anterior shoulder instability were surgically treated: 51 % (range 0–100 %).

64 % also measures the Hill Sachs lesion, to perform a remplissage [21], where 33 % is not doing (3 % is unknown). The arthroscopic labrum repair and the Latarjet procedure were the most frequent procedures performed by the respondents (respectively 94 and 54 %). To determine the most optimal treatment regime, 50 % of respondents used the ISIS [2, 23] guidelines, 32 % is not using guidelines and the other 18 % of respondents used personal experience, plain patient characteristics, percentage of bone defects and Stanmore guidelines. Based on a median of 25 % (rang 10–85 %) bone loss of the glenoid, the respondents changed their approach from soft tissue repair into a bony procedure.

Of the responding surgeons, 61 % have trained or are planning to perform an arthroscopic Latarjet procedure in future, 8 % is already performing it.

## Discussion

An international survey on clinical management of anterior shoulder instability was held at the ESSKA congress 2014. The aim of the survey was to evaluate opinions of specialist in shoulder surgery on clinical decision-making when facing patients with anterior shoulder instability. Following our hypothesis, the survey showed a wide variation in clinical practice in patients suspected of having anterior shoulder instability. Just over half of the respondents of our survey (55 %) believe that sufficient knowledge on the decision method for an operative treatment of shoulder instability is available in current literature and guidelines.

Many clinicians believe that assessing stability of the shoulder is challenging because of the complexity of the combined motions of degree of the glenohumeral and scapulothoracic joints [1]. Based on our survey, type of profession, type of sports practiced, prior shoulder trauma and number of dislocations were seen as most helpful anamnestic characteristics in guiding treatment strategy. van Kampen et al. [27] concluded that the most important predictors for recurrence were young age, previous shoulder dislocation and a sudden onset of complaints. A more recent meta-analysis [18] showed that sex, age at initial dislocation, time from initial dislocation, greater tuberosity fractures and hyperlaxity were risk factors with high evidence for recurrent instability.

A variability of clinical tests has been described to identify glenohumeral translation (determine laxity) or to provoke recurrence of the symptoms of glenohumeral instability (provocation tests) [24]. Our survey shows that the apprehension test is the most frequently used provocation test (91 %). The accuracy of shoulder instability tests are, according to a review of Luime et al. [16], not high; a solely apprehension test was found to be of limited value. Combining the apprehension test with the relocation test and the anterior release test gives the highest accuracy. The study of Lo et al. examined three different provocation tests on patients with possible (anterior) shoulder instability [13]. Out of the three tests, the surprise test had the highest accuracy. In patients who had a feeling of apprehension during the apprehension-, relocation- and surprise test, the mean positive and negative predictive values were 94 % and 72 %, respectively [13]. The study of van Kampen et al. [27] found an overall accuracy that varied between 80 and 88 % (apprehension 82 %, relocation 85 %, release 86 %, hyperabduction 81 %).

Our survey showed an average of 51 % of patients having shoulder instability undergoing surgical treatment. It was beyond the reach of our survey that the surgeons who filled out the questionnaire probably treat different populations; e.g. athletes, elderly patients. Nevertheless, a review of Longo et al. [14] showed results favouring a surgical approach above a conservative treatment with a smaller recurrence rate after surgery. Especially young adults, with high demanding sports or job activities, seem to benefit from an early surgical treatment of shoulder instability [14].

The vast majority of 94 % of the surgeons participating in our survey perform arthroscopic labrum repair on patients with shoulder instability compared to only 12 % who perform an open Bankart procedure. Due to the manner of questioning, we cannot state that the open Bankart repair was the preferred primary treatment only for certain cases or used when there was persisting instability after arthroscopic treatment. In addition, 57 % of surgeons perform an open Latarjet procedure and 8 % perform the arthroscopic Latarjet technique. A noteworthy amount of studies have been conducted on the comparison between open and arthroscopic repair in shoulder instability [6, 9, 12]. Lately some epidemiological parameters are reviewed to be significantly associated with recurrence rate after Bankart repair [20].

On a median of 25 % bone loss of the glenoid, respondents changed their approach from soft tissue repair into a bony procedure. Though this percentage had a range of 10–85 %, showing that there is still uncertainty about this cut-off value. This is in accordance with the literature [15] that is also showing the existing uncertainty about with which size of the Bankart or Hill Sachs bone defect which procedure to perform. A possible explanation, supported by this survey, is the number of different methods available how to determine this bone loss [2, 11, 17, 19, 22, 28]. Moreover recurrence rates might not be only related to choice of surgical treatment only; it can also be the result of suboptimal interpretation of the performed diagnostic strategy, protocol, and quantifying bone loss resulting in differences in subsequent treatment. Additionally, softer parameters like patients preferences can also interfere with choice of treatment and therefore cause variation. Another consideration is that in finding best diagnostic options and subsequent therapeutic regiments in patients with shoulder instability, one should use evidence-based medicine combined with clinical experience in surgical management and the patients’ wish. Due to the lack of conclusive evidence on the management of shoulder instability, surgeons have to fall back on lower evidence levels and clinical experience, which can result in variable strategies like found in this research. This and also the way of stating the questions caused a fail in showing nuances and could have had provoked recall bias in our respondents.

Because of the high number of surgeons out of different countries that participated in our survey, we think we found evidence for adopting our hypothesis that high variety in diagnostic work-up towards treatment of shoulder instability (still) exists. Therefore, to improve international consensus and thus the diagnosing and treatment of patients with shoulder instability, we recommend more research especially on this topic. Future research has to focus on quantifying bone loss and finding the exact cut-off when to perform “bony” surgery. This should improve treatment possibilities and thus satisfaction rates with fewer recurrences in patients with shoulder instability.

## Conclusion

For assessing anterior shoulder instability, a great amount of diagnostic strategies are available. In this survey a high variety in the use of these diagnostic tools is seen amongst surgeons. Also no consensus is observed in the use of therapeutic options and with which amount of bone loss choosing the right strategy in shoulder instability. A suggested solution would be an updated international consensus for using accurate diagnostic decision tools. For this, more research on diagnostic imaging and validated values of bone loss indicating specific surgical treatment is needed.
